# Priorisierte Terminvergabe bei Neuvorstellungen: Was ist wirklich entscheidend? Vergleichende Analyse manueller Terminallokation mit automatisierten und KI-gestützten Ansätzen

**DOI:** 10.1007/s00393-024-01550-7

**Published:** 2024-08-16

**Authors:** Stefan Krämer, A. Flöge, S. Handt, F. Juzek-Küpper, K. Vogt, J. Ullmann, T. Rauen

**Affiliations:** Medizinische Klinik II für Nieren- und Hochdruckkrankheiten, rheumatische und immunologische Erkrankungen, Uniklinik der RWTH Aachen, Aachen, Deutschland

**Keywords:** Terminallokation, Rheumatologische Erkrankungen, Triage, Künstliche Intelligenz (KI), Vorhersagewert, Appointment allocation, Rheumatic diseases, Triage, Artificial intelligence (AI), Predictive value

## Abstract

**Hintergrund:**

Die zeitnahe Terminvergabe für Neuvorstellungen ist eine tägliche Herausforderung in der rheumatologischen Praxis, die von digitalen Lösungen unterstützt werden kann. Es stellt sich die Frage nach einer möglichst einfachen und effektiven Methode der Terminpriorisierung.

**Methoden:**

Mithilfe eines Anmeldeformulars für Neuvorstellungen wurden standardisiert Symptome und Laborbefunde erfasst. Die Terminvergabe erfolgte nach fachärztlicher Sichtung dieser Informationen in 3 Kategorien: (a) < 6 Wochen, (b) 6 Wochen bis 3 Monate und (c) > 3 Monate. Die Wartezeiten zwischen dem Zeitpunkt der Anmeldung und dem Vorstellungstermin wurden berechnet und verglichen zwischen Patienten mit und ohne Diagnose einer entzündlich-rheumatischen Erkrankung (ERE). Zusätzlich wurde ein Entscheidungsbaum, eine Methode aus dem Bereich des überwachten Lernens innerhalb der künstlichen Intelligenz (KI), erstellt und die resultierende Klassifikation bezüglich Trefferrate und berechneter Wartezeitersparnis verglichen.

**Ergebnisse:**

Insgesamt wurden 800 Fälle (darunter 555 Frauen [69,4 %], medianes Alter 53 Jahre [IQA 39–63]) zwischen 2020 und 2023 ausgewertet. Eine ERE konnte in 409 (51,1 %) Fällen bestätigt werden mit einer Wartezeit von 58 vs. 93 Tagen bei Non-ERE-Fällen (−38 %, *p* < 0,01). Eine KI-Stratifizierung ergab eine Trefferrate von 67 % bezüglich einer ERE und eine prognostizierte Einsparung von 19 % Wartezeit. Die Trefferrate stieg hierbei auf 78 % mit einer Zeitersparnis für ERE-Fälle um bis zu 31 %, wenn grundlegende Laborergebnisse bekannt waren. Andererseits ergaben vereinfachte Algorithmen z. B. durch eine reine Laborwert-basierte Stratifizierung eine niedrigere Trefferrate und Zeitersparnis.

**Schlussfolgerung:**

Die fachärztliche Terminzuweisung ist effektiv und verkürzt die Terminwartezeit für Patienten mit ERE signifikant. Eine automatisierte Kategorisierung kann unter Berücksichtigung vollständiger Laborwerte mit reduzierter Sensitivität zu einer Verkürzung der Terminwartezeit führen.

## Abstract (en)

**Background:**

The timely allocation of appointments for new patients is a daily challenge in rheumatological practice, which can be supported by digital solutions. The question is to find the simplest and most effective possible method for prioritization when allocating appointments.

**Methods:**

Using a registration form for new patients, standardized symptoms and laboratory results were collated. After reviewing this information by a medical specialist the allocation of appointments was carried out in three categories: a) < 6 weeks, b) 6 weeks up to 3 months and c) > 3 months. The waiting time between the time of registration and the presentation appointment was calculated and compared between patients with and without a diagnosis of an inflammatory rheumatic disease (IRD). In addition a decision tree (DT), a method taken from the field of supervised learning within artificial intelligence (AI), was established and the resulting classification was compared with respect to the accuracy and calculated saving in waiting time.

**Results:**

In this study 800 appointments between 2020 and 2023 (including 555 women, 69.4%, median age 53 years, interquartile range, IQR 39–63 years) were analyzed. An IRD could be confirmed in 409 (51.1%) cases with a waiting time of 58 vs. 93 days for non-IRD cases (−38%, *p* < 0.01). An AI-based stratification resulted in an accuracy of 67% for IRD and a predicted saving of 19% waiting time. The accuracy increased up to 78% with a time saving for IRD cases of up to 31%, when all basic laboratory results were known. Simplified algorithms, e.g., stratification by the use of laboratory findings alone, resulted in a lower accuracy and time savings.

**Conclusion:**

Manual allocation of appointments by a medical specialist is effective and significantly reduces the waiting times for patients with IRD. An automated categorization can lead to a reduction in waiting times for appointments when taking complete laboratory results and a lower sensitivity into consideration.

## Hinführung

Vor dem Hintergrund des fachärztlichen Versorgungsengpasses in weiten Teilen Deutschlands ist die Einplanung von Neuvorstellungen eine erhebliche Herausforderung in der rheumatologischen Praxis. In den letzten Jahren sind mehrere digitale Anmeldeplattformen wie RhePort/Rhadar [7] oder Rheuma-VOR [13] zur Kategorisierung von Neupatienten in mehreren Bundesländern erfolgreich etabliert worden, die eine detaillierte Symptomeingabe durch Patienten oder zuweisende Ärzte mittels App oder Internetportal erfordern. Für uns stellte sich die Frage, welche Ergebnisse – speziell hinsichtlich der Wartezeit – unter Berücksichtigung weniger Parameter, die im Rahmen der Routineanmeldung erfasst werden können, zu erwarten sind. Diese Parameter könnten sich in Standardportale mit künstlicher Intelligenz (KI)-unterstützter Terminvergabe integrieren lassen.

## Hintergrund

Zahlreiche aktuelle Empfehlungen nationaler und internationaler Fachgesellschaften sehen einen rheumatologischen Facharzttermin beim Verdacht auf eine rheumatoide Arthritis binnen 6 Wochen vor [2, 12]. Um eine zeitgerechte Versorgung zu gewährleisten, wird ein Verhältnis (Facharztstelle auf Einwohner) von 1:50.000 gefordert. Bei deutschlandweit 1,5 Mio. Patienten mit einer entzündlich-rheumatischen Erkrankung (ERE) resultiert hieraus ein Bedarf von 1340 Rheumatologen [3, 21]. Insgesamt wurden multiple Anstrengungen zur Verkürzung der langen Wartezeiten für eine rheumatologische Abklärung vorgeschlagen z. B. durch Etablierung von Frühsprechstunden [1, 3]. Letztlich bedürfen auch diese Sprechstunden einer gewissen Vorselektion, sofern man keine komplett offene Sprechstunde ohne Terminvergabe einrichten möchte [6, 17].

Es wurden bereits etliche digitale Systeme zum Screening von Rheumapatienten und auch zur Terminvergabe vorgestellt und umfangreich evaluiert [8, 11, 16]. Hierbei wird eine teils aufwendige Symptomenabfrage vorausgesetzt, und es ergibt sich die Frage, ob auch durch vereinfachte Abfragen eine Terminpriorisierung möglich ist, um diese auch in konventionelle Online-Terminvergabeplattformen integrieren zu können. In der aktuellen Arbeit sollen erforderliche Grundparameter näher untersucht und eine manuelle Vergabestrategie als Vergleich evaluiert werden.

In der Städteregion Aachen leben etwa 470.000 Erwachsene [10]. Diese sollten im Idealfall gemäß dem von der DGRh vorgeschlagenen Versorgungsschlüssel (2 Rheumatologen pro 100.000 Einwohner) von 9,4 ambulant rheumatologisch tätigen Ärztinnen und Ärzten versorgt werden. De facto waren jedoch im Jahr 2022 nur 2 rheumatologische KV-Sitze und 2 weitere Sitze über eine Krankenhaus-gebundene Ermächtigungsambulanz für die ambulante Versorgung abgebildet. Daraus ergab sich ein Versorgungsschlüssel von deutlich weniger als einem Rheumatologen pro 100.000 erwachsene Einwohner, was deutlich weniger als 50 % der angestrebten Quote entspricht. Daher haben wir uns entschlossen, Vorstellungstermine für Neupatienten zur routinemäßigen rheumatologischen Abklärung in der 2019 neu eingerichteten rheumatologischen Hochschulambulanz über den Zeitansatz zu steuern. Dabei haben wir uns an einem durchschnittlichen Zeitansatz von maximal 3 Monaten bis zum Vorstellungstermin orientiert. Bei hoher Wahrscheinlichkeit für eine ERE und/oder hoher Entzündungslast sollte ein früher Termin (unter 6 Wochen) möglich sein.

Als Grundlage zur Datenerhebung diente ein von den zuweisenden niedergelassenen Ärzten übermitteltes Anmeldeformular mit Angaben zu wichtigen Symptomen und Laborwerten (einschließlich C‑reaktives Protein [CRP], Blutsenkungsgeschwindigkeit [BSG], Rheumafaktor [RF] und CCP-Autoantikörpern [Anti-CCP]). Weitere Unterlagen wie Laborausdrucke, Bildgebungsbefunde usw. konnten als Anlage beigefügt werden.

### Ziele.

In einem ersten Schritt erfolgten die Sichtung und Terminzuordnung durch einen erfahrenen Facharzt für Rheumatologie. Die Auswertung des ersten Abschnittes ergab bereits eine signifikante Verkürzung in der Wartezeit um 3 Wochen für diejenigen Fälle, die sich retrospektiv als ERE (gegenüber Nicht-ERE-Fällen) erwiesen [9].

In der vorliegenden Arbeit sollte weitergehend untersucht werden:

- auf welche wesentlichen Parameter sich diese effektive Terminkategorisierung stützt,
- ob und wie eine Automatisierung der Triage von Standardfällen mit Unterstützung von künstlicher Intelligenz (KI) möglich ist und
- welche quantitativen Ergebnisse, z. B. in Form von Zeitersparnis, durch einfache Modelle zu erwarten sind.

Ein wesentlicher Fokus der Studie liegt in der Nachvollziehbarkeit und Bewertung der herangezogenen Kriterien. Dabei wird die Bewertung der Parameter durch klassische Statistik (z. B. logistische Regression) denen der KI (z. B. im Entscheidungsbaum) gegenübergestellt und diskutiert.

## Material und Methoden

Es wurden alle Anmeldungen zur Erstvorstellung für einen rheumatologischen Termin in der Hochschulambulanz am Universitätsklinikum der RWTH Aachen zwischen November 2020 und Oktober 2023 gesammelt. Die Auswertung erfolgte nach positivem Ethikvotum der RWTH (21-052) anhand eines papierbasierten, von den Zuweisern (zu ca. 84 % aus der Allgemeinmedizin) in der Regel per Fax übermittelten Anmeldeformulars mit Angaben zu Alter, Geschlecht, Freitextangaben zur Verdachtsdiagose und Symptomen sowie Eingabezeilen für RF, Anti-CCP, CRP, BSG, antinukleäre Antikörper (ANA) und HLA(„human leukocyte antigen“)-B27. ANA wurden ab einem Titer von 1:80, CRP ab 5 mg/l als positiv gewertet. Zusätzliche Labor- oder Bildgebungsbefunde konnten fakultativ angefügt werden. Neben den Laborparametern wurden – sofern angegeben – auch die übermittelten Leitsymptome (Gelenkschmerz, -schwellung oder -steifigkeit, Rückenschmerz, Raynaud-Syndrom und Fieber) in die Datenbank aufgenommen. Nach fachärztlicher Sichtung der Anmeldungsformulare wurde eine Terminkategorie mit vordringlicher (< 6 Wochen), normaler (6 Wochen bis 3 Monaten) oder nachrangiger (> 3 Monate) Priorität vergeben. Nach erfolgtem Vorstellungstermin wurden der Termin sowie die gestellte Diagnose als Grundlage der Auswertung erfasst.

## Elementare Auswertung und Statistik

In der Auswertung wurden die Diagnosen den übergeordneten Kategorien „ERE“ oder „Nicht-ERE“ zugeordnet, und der zeitliche Abstand zwischen Anmeldung des Patienten und tatsächlichem Termin (in Tagen) wurde ermittelt. Die Alters- und Geschlechterstruktur wurde grafisch dargestellt. Die Unterschiede in der Wartezeit zwischen den Kategorien wurden berechnet und mittels ANOVA-Tests verglichen. Der Einfluss der quantitativ (CRP und BSG) und qualitativ erfasster Parameter (RF, Anti-CCP, ANA, HLA-B27) auf die Diagnose wurde mittels logistischer Regression untersucht.

## Maschinelles Lernen („machine learning“ [ML])

Anhand der vorhandenen Kategorisierung wurde in mehreren Schritten die Wertigkeit der qualitativen und quantitativen Anmeldungsdaten für die spätere Klassifikation als „ERE“ oder „Nicht-ERE“ in einem dichotomen Entscheidungsbaum analysiert. Hierbei handelt es sich um ein Grundverfahren aus dem überwachten, maschinellen Lernen („supervised ML“), bei dem mithilfe eines Optimierungsverfahrens durch geeignete Verzweigungen immer kleinere Gruppen (Blätter) mit immer homogenerem Inhalt gebildet werden. Zum Einsatz kam hier die Python (Python 3.1)-Bibliothek scikit-learn (V 1.3.2) [14], bei der nach dem Gini-Kriterium optimiert wurde. Der Vorteil in diesem Verfahren liegt in der grafischen Nachvollziehbarkeit der Kriterien, die zur Klassifikation führen. Wie bei Klassifikationsmethoden im ML üblich, wurde ein Teil (hier 60 %) der Ausgangsdaten zum Training verwendet und der Entscheidungsbaum anhand der verbliebenen 40 % validiert. Ein Austausch des KI-Algorithmus ist auf Mailanfrage über den korrespondierenden Autor möglich. Zunächst wurde die Trefferrate („accuracy“) des Algorithmus in Abhängigkeit der eingespeisten Daten verglichen, definiert als Verhältnis zwischen richtig (als ERE) zugeordneten Fällen und der Gesamtzahl der Fälle. Zusätzlich wurden die erstellten Klassifikationen auf den gesamten Datensatz übertragen, und eine Simulation der Wartezeiten wurde errechnet und ausgewertet. Die deskriptive und schließende Statistik sowie die Grafiken wurden mithilfe des Statistikprogramms R bzw. RStudio (posit software, Boston, Massachusetts, USA, Version 2023.03.0 386) und des entsprechenden Python-Pendants (matplotlib) erstellt.

## Ergebnisse

**Abb. 1:**
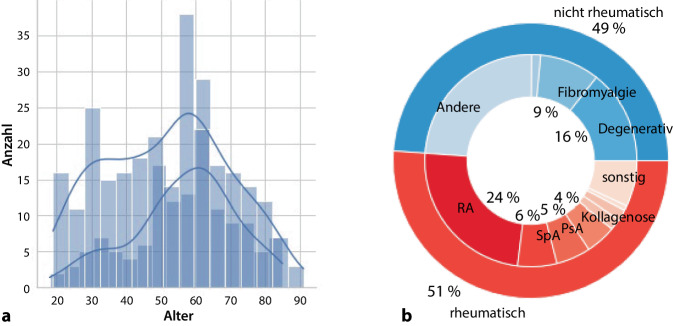
Bestätigte ERE-Fälle nach Alter und Geschlecht (**a**) sowie ermittelten Diagnosen (**b**). Weiblich: *hellblaue Balken/Kurve*, männlich: *dunkelblaue Balken/Kurve*; *RA* rheumatoide Arthritis, *SpA* Spondylarthritis, *PsA* Psoriasisarthritis

Auswertbar waren 800 Datensätze von Patienten, deren Anmeldung zur Neuvorstellung in der Hochschulambulanz am Universitätsklinikum Aachen zwischen November 2020 und Oktober 2023 erfolgte. Das mediane Alter betrug 53 Jahre (Interquartilsabstand: 39 bis 62 Jahre), 555 Patienten waren weiblichen Geschlechts (69,4 %). Die Altersstruktur aller Fälle zeigt im geglätteten Histogramm eine geschlechterunabhängige, zweigipflige Kurve (Abb. 1a). In der Gesamtkohorte waren Männer mit bestätigter Diagnose einer ERE mit 63 Jahren etwas älter (vs. 59 Jahren bei Frauen, *p* < 0,01, Tab. 1). Der Altersunterschied ist im Wesentlichen im Falle der rheumatoiden Arthritis erkennbar, bei der Frauen im Durchschnitt um 3 Jahre jünger waren (*p* < 0,02).

**Tab. 1:** Diagnoseverteilung der Neuvorstellungen nach Untersuchung

	*n* (%)	*n* (%)	Alter^a^ [J, IQA]	*n* (%)	Alter^a^ [J, IQA]	*p*-Wert^a^
–	*N* = 800	Weiblich		Männlich		–
		555 (69,4)	53 (37–67)	245 (30,6)	54 (42–64)	n. s.
*ERE*	409 (51,1)	265 (47,7)	53 (36–65)	144 (58,8)	59 (48–66)	< 0,01
Rheumatoide Arthritis	193 (24,1), 47 %-Anteil^b^	138 (24,9), 52 %-Anteil	57 (40–66)	56 (22,9), 39 %-Anteil	60 (53–68)	0,02
Psoriasisarthritis	42 (5,3), 10 %-Anteil	20 (3,6), 8 %-Anteil	49 (42–57)	22 (9), 15 %-Anteil	53 (44–62)	n. s.
*Nicht-ERE*	391 (48,9)	290 (52,3)	53 (39–60)	101 (41,2)	47 (36–61)	n. s.
Fibromyalgie	73 (9,1), 19 %-Anteil^c^	63 (11,4), 22 %-Anteil	50 (38–57)	10 (4,1), 10 %-Anteil	52 (45–55)	n. s.
Degenerativ	115 (14,4), 29 %-Anteil	91 (16,4), 31 %-Anteil	58 (53–68)	24 (9,8), 24 %-Anteil	55 (42–62)	0,05

^a^ Signifikanzniveau des Altersvergleichs der Geschlechter

^b^ Anteil an ERE

^c^ Anteil an Nicht-ERE

Als häufigste Laborwerte wurden CRP, RF und Anti-CCP von den Zuweisern übermittelt. Hauptsächlich berichtete Symptome waren Gelenkschmerzen, eine Übersicht über die Angabe weiterer Symptome gibt Tab. 2. Nach erfolgter Diagnose erwiesen sich 51,1 % als ERE, darunter entfielen die meisten Fälle auf eine rheumatoide Arthritis. Spondylarthritiden und Psoriasisarthritiden folgten in größerem Abstand (Abb. 1b). Unter den Nicht-ERE-Fällen machten degenerative Erkrankungen gefolgt von Fibromyalgie die Mehrzahl der diagnostizierbaren Fälle aus, wobei etwas weniger als die Hälfte der Nicht-ERE-Fälle in Ausschlussdiagnosen resultierten (als „andere“ ausgewiesen).

**Tab. 2:** Übermittelte Parameter für Neuvorstellungen

Parameter	Positiv	Negativ	Nicht berichtet
CRP	156 (19,5)	239 (29,9)	405 (50,6)
BSG	85 (10,6)	161 (20,1)	554 (69,3)
RF	93 (11,6)	206(25,8)	501 (67,6)
Anti-CCP	69 (8,6)	201 (25,1)	530 (66,3)
HLA-B27	22 (2,8)	9 (1,1)	769 (96,1)
ANA	103 (12,9)	33 (4,1)	664 (83)
ANCA	6 (0,8)	16 (2)	778 (97,3)
*Symptome*			
Schmerzen	516 (64,5)	Raynaud	23 (2,9)
Schwellung	136 (17)	Rückenschmerzen	65 (8,1)
Steifigkeit	16 (2)	Hautveränderungen	37 (4,6)

## Prädiktiver Werte von Symptomen und Laborwerten

**Abb. 2:**
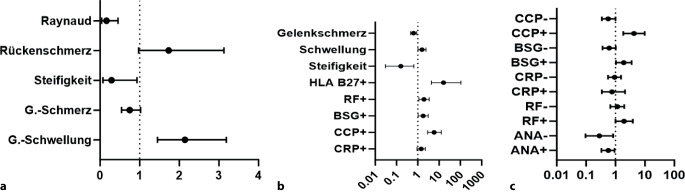
Multivariate logistische Regression der Anmeldeparameter. **a** Symptome, **b** Symptome und Laborbefunde nur wenn positiv (+), **c** Laborparameter mit positiver vs. negativer (−) Ausprägung im Vergleich. Die Lage < 1 korreliert negativ, > 1 positiv mit der Diagnose einer rheumatischen Erkrankung. *G* Gelenk

Den höchsten Stellenwert für die Diagnose einer ERE ergab sich in der univariaten Analyse für Angabe einer Gelenkschwellung (OR 1,6; 95 %-KI 1,12–2,32; *p* = 0,012), andere Befunde waren nicht signifikant positiv korreliert. Die multivariate Auswertung der Symptome ist in Abb. 2a wiedergegeben.

Die univariate Analyse der Laborparameter ergab die höchste Wahrscheinlichkeit für das Vorliegen einer ERE für das Vorhandensein des Marker HLA-B27 mit einer Odds-Ratio (OR) von 10,0 (95 %-KI 3–9; *p* = 0,002), wobei hierzu anzumerken ist, dass in knapp 4 % der Fälle eine HLA-B27-Bestimmung im Vorfeld vorlag. Im Weiteren zeigten sich das Vorliegen von Anti-CCP (OR 6,4; 95 %-KI 3,4–13,5; *p* < 0,001), des Rheumafaktors (OR 2,8; 95 %-KI 1,73–4,5, *p* < 0,001) und eines erhöhten CRP (OR 1,81; 95 %-KI 1,27–2,6, *p* = 0,001) als prädiktiv für die Diagnose einer ERE. ANA-Positivität war mit einer OR von 0,51 (95 %-KI 0,33–0,78, *p* = 0,002) genauso negativ korreliert wie ein bekannt negativer ANA-Titer (OR 0,34; 95 %-KI 0,15–0,72; *p* = 0,007). Die multivariate Auswertung ist der Abb. 2b zu entnehmen.

Betrachtet man positive und negative Laborbefunde gleichzeitig in einer Analyse (Abb. 2c), so ergeben sich für die meisten Parameter gegensätzliche Wahrscheinlichkeiten, am stärksten bei den Anti-CCP-Antikörpern mit sowohl positiver als auch negativer signifikanter Korrelation. Dieser Effekt ist in unserer Kohorte für die BSG noch deutlich stärker als für den CRP-Wert, für ANA gilt dieses Verhalten nicht.

## Wartezeitanalyse

Patentenfälle, die sich nach Vorstellung als ERE herausstellten, hatten eine mittlere Terminwartezeit von 58 Tagen bis zum Vorstellungstermin, während Nicht-ERE-Fälle 93 Tage warteten. Die Differenz betrug 35 Tage (also minus 38 %; *p* < 0,01) für die Kategorisierung/Terminzuweisung nach fachärztlicher Sichtung („reales Modell“).

## Modellierung der Terminvergabe

**Abb. 3:**
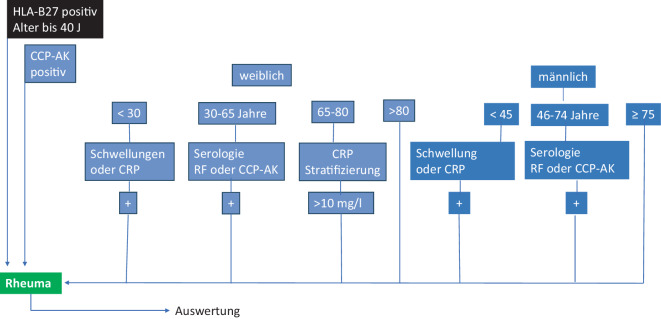
Durch einen einfachen Entscheidungsbaum mit wenigen Ebenen inspirierter Algorithmus

- **Einfaches KI-Modell:** Unter der Vorgabe nur weniger (≤ 4) Unterscheidungsstufen wurde anhand der Kriterien, die sich im Trainingsdatensatz fanden, ein einfacher Entscheidungsbaum entsprechend Abb. 3 entworfen.
- **Komplexes KI-Modell **(mit tiefem Entscheidungsbaum): Hierbei wurde ein komplexes Modell mit allen Parametern trainiert, welches bis 8 Unterscheidungsstufen unterteilen konnte.
- **Labormodell: **Vergleichsmodell mit einer rein auf Laborwerten basierten Kategorisierung, die alle Fälle als „wahrscheinliche“ ERE ansah und damit einen dringlichen Vorstellungstermin vorschlug (binnen 6 Wochen), welche mindestens eines der folgenden Kriterien aufwies: RF-, Anti-CCP-, HLA-B27-Positivität, CRP oder BSG positiv. Alle anderen Fälle wurden als „ERE unwahrscheinlich“ eingestuft.

Für die 3 Modelle sind die Trefferquoten für das Vorliegen einer ERE, Sensitivitäten, Spezifitäten und Unterschiede der Wartezeiten zwischen ERE- und Nicht-ERE-Fällen in Tab. 3 dargestellt. Dabei wurden allen Fällen des Datensatzes, die im entsprechenden Modell als ERE angesehen wurden, einer Wartezeit von 60 Tagen (dem ungefähren Mittelwert des „realen Modells“ entsprechend) und allen als „Nicht-ERE“ klassifizierten der doppelte Werte (120 Tage) zugeordnet, welches der Zielvorgabe einer Vorstellung in einem Zeithorizont länger als 3 Monate bei der manuellen Vergabe in der Realität nahekommt.

**Tab. 3:** Ergebnisse verschiedener Klassifikationsmethoden

Verfahren	*n*, Rheuma ja/nein	Trefferrate (in %)	Sensitivität (in %)	Spezifität (in %)	Wartezeit [Tage]	Delta	*p*
Reales Modell (nach FA-Sichtung)	800, 409/391	n. a.	n. a.	n. a.	58 vs. 93	35 (38 %)	< 0,01
Einfaches KI-Modell^a^	800	63	47	77	93 vs. 106	13 (12 %)	< 0,01
Komplexes KI-Modell^c^	800	67	47	89	92 vs. 113	21 (19 %)	< 0,01
Labormodell^b^	800	62	43	78	96 vs. 106	10 (9 %)	< 0,01
*KI,-Modell, für den Fall, dass ...*							
CRP und RF bekannt	229, 117/112	75	57	94	86 vs. 116	30 (26 %)	–
CRP, RF und CCP bekannt	179, 92/87	78	64	93	82 vs. 116	34 (29 %)	
CRP, RF, CCP und BSG bekannt	95, 62/33	78	76	81	75 vs. 109	34 (31 %)	

*FA* Facharzt

^a^ Analog Abb. 3

^b^ Falls mindestens 1 Parameter aus: HLA-B27, Anti-CCP, RF oder CRP erhöht vorliegt, als ERE gewertet

^c^ Entscheidungsbaum aus allen vorliegenden Parametern

Im komplexen KI-Modell (mit tiefem Entscheidungsbaum) wurde mit 67 % die höchste Trefferrate für das Vorliegen einer ERE ermittelt, die sich nochmals bis auf maximal 78 % verbesserte, wenn tatsächlich alle Laborbefunde (CRP, BSG, RF und Anti-CCP) vorlagen. Dieser Zugewinn resultierte v. a. aus einer gestiegenen Sensitivität. Entsprechend reduzierte sich auch die Wartezeit für Patienten mit ERE im Vergleich zu Nicht-ERE-Fällen um bis zu 31 %, wenn alle Laborwerte im Vorfeld von den Zuweisern übermittelt wurden. Eine reine Stratifikation auf Basis von Laborwerten („Labormodell“) erbrachte hier die geringste Trefferquote und theoretisch ermittelte Zeitersparnis.

**Abb. 4:**
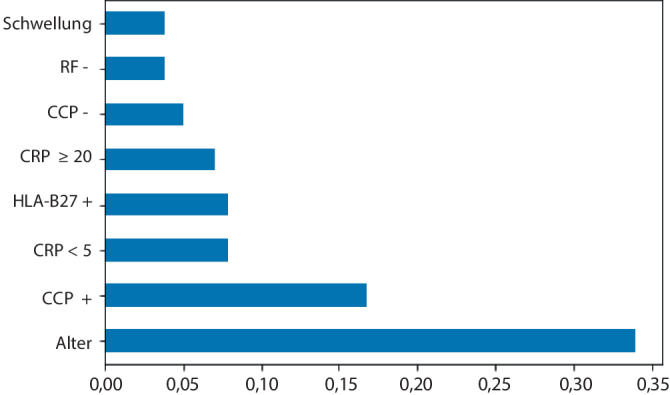
Informationsgewinn durch einzelne Parameter (Gini-Index) für die Klassifikation ERE vs. Nicht-ERE im komplexen Entscheidungsbaum, dargestellt als Anteile von 1 für alle Fälle mit bekanntem Status für RF und CCP-Antikörper

Den Stellenwert einzelner Parameter bei zumindest bekanntem Befund für RF und CCP in den Anmeldebefunden nach Entscheidungsbaum mittels Gini-Index gibt Abb. 4 wieder. Dabei wurden CRP-Werte als Kategorien der Stufen von negativ (< 5) über jeweils ≥ 5, 10, 20, 30, 40 und 50 mg/l in die Bewertung mit aufgenommen. Im Diagramm entspricht die Länge des Balkens dem Anteil am gesamten Informationsgewinn. Der hierbei relevanteste Parameter entspricht nicht notwendigerweise der ersten (und somit wichtigsten) Aufzweigung des Baumes, da Parameter ggf. mehrfach auftreten und damit stärker zur gesamten Aufklärung beitragen. Hierbei entfallen ca. 17 % auf einen positiven CCP-Antikörper, gut 15 % auf den Parameter CRP als Summe für negativ und für ≥ 20 mg/l angegebene Befunde. Der Rheumafaktor geht hier nur bei negativem Befund in die Bewertung ein. Der auffällig hohe Wert des Parameters „Alter“, der > 30 % der Entscheidungsfindung im Modell ausmacht, erscheint nur deshalb plausibel, da er als einziger der quantitativen Größe ausnahmslos als metrische Größe vorlag. Ein positiver HLA-B27-Status bei Anmeldung weist die höchste Wahrscheinlichkeit für das Vorhandensein einer rheumatischen Erkrankung gemäß Abb. 2b auf, der Parameter tritt hier aber nur im Mittelfeld (um 7 %) in Erscheinung, da er nur in einem sehr kleinen Anteil (3,9 %, Tab. 1) übermittelt wurde bzw. auch nur bei spezieller Fragestellung relevant ist.

## Diskussion

Die Rate an ERE in unserer rheumatologischen Hochschulambulanz rangiert mit 51,1 % aller Neuvorstellungen in der oberen Spanne vergleichbarer Erhebungen, bei denen zwischen 40 und 52,8 % der Erstvorstellungen entzündlich-rheumatische Erkrankungen aufwiesen [4]. Im niedergelassenen Versorgungsbereich dürfte die Rate deutlich niedriger liegen. Unter den ERE-Fällen fanden sich prädominant Fälle mit rheumatoider Arthritis, Psoriasisarthritis und Spondylarthritiden. Eben diese Diagnosen wurden auch in bisherigen Versorgungsmodellen wie Rheuma-VOR und Registerdatensätzen des Dt. Rheumaforschungszentrums (DRFZ) und des Berufsverbandes (BDRh) fokussiert untersucht [13, 20]. Insofern kann die vorliegende Arbeit als repräsentativ angesehen werden.

Die fachärztliche Sichtung von Vorunterlagen mit entsprechender Einbestellung zeigt einen signifikanten Vorteil für die Patienten, bei denen letztlich eine ERE diagnostiziert wurde, im Sinne einer um 38 % kürzeren Wartezeit bis zum Vorstellungstermin, nämlich im Mittel um 35 Tage gegenüber Patienten, bei denen keine ERE festgestellt wurde. Hierfür muss jedoch ein nicht unerheblicher, hier nicht weiter quantifizierter, zeitlicher Aufwand betrieben werden. Der von uns gewählte, komplexe KI-basierte Ansatz erreicht für Trefferrate bzw. Zeitersparnis vergleichbare Dimensionen wie die fachärztliche Sichtung, sofern tatschlich alle Laborparameter sowie einige Schlüsselsymptome vorliegen. Hier fanden wir, dass sich erwartungsgemäß mit zunehmender Anzahl an verfügbaren Laborwerten die Sensitivität im Modell deutlich verbesserte, wohingegen die Spezifität primär von einer höheren Komplexität des KI-Modells profitierte. Im Gegensatz dazu führt eine rein auf Laborwerte abzielende Triage zu deutlich unterlegenen Ergebnissen, dennoch ist auch hierfür ein signifikanter Effekt nachweisbar, der sich im theoretischen Modell auf errechnete 10 Tage (9 %) Zeitersparnis beläuft. Diese Stratifizierung stellt im Vergleich zu den etablierten digitalen Anmeldelösungen (z. B. RhePort/Rhadar) eine vereinfachte Option der Patientensteuerung im Spektrum der Möglichkeiten dar [8, 19].

Bei der Analyse des Stellenwertes einzelner Laborwerte lässt sich erkennen, dass die Nutzung des ANA-Titers als Screening für ERE gänzlich ungeeignet erscheint und somit im hausärztlichen Setting ohne konkreten klinischen Verdacht einer Kollagenose nicht genutzt werden sollte. Diese Resultate werden auch durch vorhandene Untersuchungen an gesunden Kohorten unterstützt, die bei bis zu 2,5 % der Allgemeinbevölkerung einen positiven ANA-Titer erheben konnten [5, 18]. Ebenfalls wurde bereits von einem unkritischen Einsatz eines ANA-Suchtestes zur Abklärung unspezifischer Gelenkbeschwerden in der Orthopädie abgeraten [15].

## Limitationen der Studie

Einschränkend sollte betont werden, dass dieses Anmeldungsmodell für elektive Routinefälle angewendet wurde und nicht für rheumatologische Notfälle, die binnen Tagen gesehen werden müssen. Daher erscheinen in den Statistiken Vaskulitiden (auch die Riesenzellarteriitis als „klassischer rheumatologischer Notfall“) und Kollagenosen stark unterrepräsentiert. Diese Patienten machen aber einen durchaus relevanten Anteil unter den Fällen in einer rheumatologischen Hochschulambulanz aus. Die Erstdiagnose erfolgte hierbei häufig während eines stationären Aufenthaltes. Auch ist eine direkte Vergleichbarkeit mit Programmen, die als rheumatologische „Symptom-Checker“ direkt von Patienten genutzt werden können [7], eingeschränkt. Durch die Filterfunktion der vorgeschalteten Instanz (in der Regel die Hausarztpraxis) unserer Untersuchung erklärt sich die hier deutlich höhere Quote an rheumatischen Diagnosen (gut 50 % vs. einem Drittel) durch die erhöhte Vortestwahrscheinlichkeit.

Nicht bei allen Neuanmeldungen wurden uns von den Zuweisern alle Informationen übermittelt, wobei sich die Anwendung der KI nachweislich verbesserte, wenn wir auf vollständige Datensätze zugreifen konnten. Anderseits sind die Angaben, sofern vorhanden, in der Regel valide, da sie durch medizinisches Personal eingetragen wurden. Hier könnte ein Nachteil bei patientenseitigem Vorgehen der Online-Portale liegen, die ihren Schwerpunkt naturgemäß auf die Symptomausdifferenzierung ausgerichtet haben. Die in unserer Erhebung berücksichtigten Symptome stellen hier nur Oberkategorien da, die sich teilweise als nicht zielführend erwiesen haben. Zwar war beispielsweise das Symptom der Gelenkschwellung gut mit dem Vorliegen einer ERE assoziiert, für das ebenfalls nützliche Symptom der Steifigkeit galt dies hingegen nicht. Ein Erklärungsansatz könnte hier in der nicht berücksichtigten Dauer bzw. der Konsistenz der Steifigkeit liegen, da auch bei Fibromyalgie und Arthrose eine gewisse Morgensteifigkeit angegeben wird.

## Ausblick

Eine recht praktikable Zwischenlösung, welche die Zuweiserpraxen entlasten und trotzdem eine adäquate und teilweise automatisierte Terminsteuerung erlauben, könnte neben der Implementation bestimmter Parameter in Online-Terminvergabeportale auch in einfachen elektronischen Direktanmeldungen über die (eigene) Webseite, die über einfache Formulare die Informationen patientenseitig mittels Pflichtfeldern abfragt und diese ggf. bereits mit Kategorisierung an den Empfänger weiterleitet, liegen.

## Fazit für die Praxis

- Patienten, die unter Angabe einiger weniger klinischer und laborchemischer Angaben durch die zuweisenden Kollegen in einer rheumatologischen Hochschulambulanz vorgestellt werden, erhalten nach fachärztlicher Befundsichtung signifikant früher einen Termin.
- Komplexe automatisierte Systeme erreichen ebenfalls hohe Trefferraten.
- Einfache Algorithmen sind allerdings nur wenig besser als eine reine Laborstratifizierung.
- Im Gegensatz zu CRP, BSG, HLA-B27, RF und Anti-CCP-Antikörpern sind ANA-Autoantikörper ohne konkreten Verdacht auf Kollagenose als hausärztliches Screening ungeeignet.
- Als problematisch bei automatisierten Ansätzen erweisen sich die eingeschränkte Sensitivität, unvollständige Daten und ggf. noch nicht genug ausdifferenzierte Parameter.

## Data Availability

Die erhobenen Datensätze sind in anonymisierter Form beim korrespondierenden Autor auf Anfrage verfügbar.
